# Increased Mobility of the Atrial Septum in Aortic Root Dilation: An Observational Study on Transesophageal Echocardiography

**DOI:** 10.3389/fphys.2021.701399

**Published:** 2021-08-24

**Authors:** Altair Heidemann, Lorença Dall'Oglio, Eduardo Gehling Bertoldi, Murilo Foppa

**Affiliations:** ^1^Graduate Studies Program in Cardiology, Universidade Federal do Rio Grande do Sul, Porto Alegre, Brazil; ^2^Cardiology Division, Hospital de Clínicas de Porto Alegre, Porto Alegre, Brazil; ^3^NUPIC (Núcleo de Pesquisa em Imagem Cardiovascular), Hospital de Clínicas de Porto Alegre, Porto Alegre, Brazil; ^4^School of Medicine, Universidade Luterana do Brasil, Porto Alegre, Brazil; ^5^School of Medicine, Universidade Federal de Pelotas, Pelotas, Brazil

**Keywords:** atrial septal aneurysm, aortic dilation, atherosclerosis, transesophageal echocardiography, stroke

## Abstract

**Background:** There is a growing interest in the relationship between atrial septal anatomy and cardioembolic stroke. Anecdotal reports suggest that the enlargement of the aortic root could interfere with atrial septal mobility (ASM). We sought to investigate the association between ASM and aortic root dilation.

**Methods and Findings:** From all consecutive clinically requested transesophageal echocardiogram (TEE) studies performed during the study period in a single institution, we were able to review and evaluate the ASM and anteroposterior length, aortic root diameter, and the prevalence of atrial septal aneurysm (ASA) and of patent foramen ovale (PFO) in 336 studies. Additional variables, such as left ventricular ejection fraction, left atrial diameter, diastolic dysfunction, age, sex, weight, height, previous stroke, atrial fibrillation, and TEE indication, were extracted from patient medical records and echocardiographic clinical reports. In 336 patients, we found a mean ASM of 3.4 mm, ranging from 0 to 21 mm; 15% had ASA and 14% had PFO. There was a 1.0 mm increase in ASM for every 10-mm increase in aortic root diameter adjusted for age, sex, weight, height, ejection fraction, and left atrial size (*B* = 0.1; *P* = 0.04). Aortic diameter was not associated with a smaller septal length (*B* = 0.03; *P* = 0.7).

**Conclusion:** An increased motion of the atrial septum can occur in association with aortic dilation. These findings deserve attention for the relevance of aortic root anatomy in future studies involving atrial septal characteristics and embolic stroke risk.

## Introduction

Embolic stroke has multiple causes, and more than one disease is frequently detected during the assessment of patients (Chatzikonstantinou et al., 2012; Amarenco et al., 2013). There is a growing knowledge regarding the roles of patent foramen ovale (PFO) and atrial septal aneurysm (ASA) as sources of embolism in ischemic stroke (Pearson et al., 1991; Lamy et al., 2002; Ward et al., 2006; Mas et al., 2017; Saver et al., 2017; Søndergaard et al., 2017). Other phenotypic expressions of large vessel atherosclerotic diseases, such as aortic enlargement and atherosclerotic plaques, are also frequently present in these patients, and they increase with aging (Reed et al., 1992). However, the causal role of diffuse advanced atherosclerosis and senile aortic dilation in stroke as well as their interconnections with other stroke risk factors is less understood.

The anatomic relation between the aortic root and atrial septum first appeared on case reports of patients with platypnea–orthodeoxia syndrome (Medina et al., 2001; Chopard and Meneveau, 2013; Hasegawa et al., 2020). An enlarged aorta could geometrically impinge a shortening in the anteroposterior atrial septal length, consequently augmenting the atrial septal mobility (ASM) (Supplementary Video). This effect, added to the presence of atrial septal defects or PFO, is part of the mechanism of hypoxia in this syndrome (Eicher, 2005). Moreover, differences in the left and right atrial filling pressures may influence the interatrial septum (IAS) movement dynamics.

The potential of aortic root enlargement to increase ASM has been previously suggested (Bertaux et al., 2007). This hypothesis deserves to be tested. Transesophageal echocardiogram (TEE) has high spatial and temporal resolutions, with high sensitivity for detecting PFO with the use of agitated saline solution (Di Tullio, 2010), and allows evaluation of the anatomic relationships between IAS and aortic root. In this study, we aimed to investigate the associations between aortic root and atrial septal morphological and functional characteristics in patients subjected to TEE for clinical indications.

## Methods

We identified 508 consecutive TEEs performed for clinical indications from January 2014 to December 2015 in the Hospital de Clínicas de Porto Alegre, a tertiary care teaching hospital in Brazil. The institutional review board approved this study and individual informed consent was waived due to the retrospective analysis of data.

Clinical variables, extracted from electronic medical records, were age, sex, weight, height, clinical indication for TEE, medical history of previous stroke, and atrial fibrillation. Additional echocardiographic data were extracted from transthoracic reports (performed regularly in all patients who had TEE), including left ventricular ejection fraction (LVEF), left atrial diameter, diastolic dysfunction, and central venous pressure estimate.

**Figure 1:**
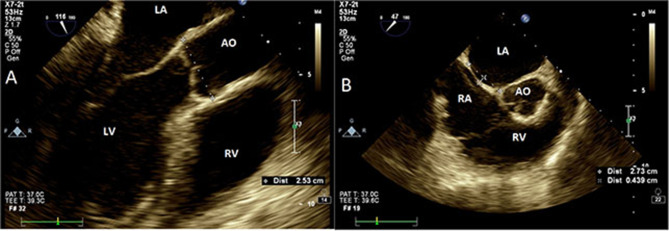
Transesophageal echocardiography. **(A)** Aortic measurements at Valsalva sinus plane (120°, mid-esophageal view). Aorta measured at leading edge echo signal of posterior wall to the leading edge of anterior wall. **(B)** Interatrial septal size and oscillation (25–45°, mid-esophageal view) measured as the maximal perpendicular distance from an imaginary line drawn between atrial septal insertion points (dashed line). AO, aorta; LA, left atrium; RA, right atrium; LV, left ventricle; RV, right ventricle.

A single investigator (AH) reviewed all archived TEE images, and study-specific measurements were performed, which were blinded to the TEE clinical reports. Aortic root size was measured from leading-to-leading edge at mid-esophageal view 120–140° at end diastole (Figure 1A). Cine loops at mid-esophageal view 25–45° at the plane of Valsalva sinus, showing both the thin component of atrial septum and the aortic root, were used to quantify atrial septal length and oscillation. ASM was defined as the distance between the maximal leftward and rightward atrial positions during spontaneous breathing (Figure 1B). Aortic plaques were computed as simple (<4 mm) or complex. Contrast study with a saline–air solution was routinely performed to identify PFO. All measurements were performed with QLab software, version 3.3.2 (Philips Healthcare, Andover, MA) and images were acquired with commercially available equipment (EPIQ7 and IE33, Philips Ultrasound, Bothell, WA) with a multiplane 7.5 MHz transducer.

Sample size was estimated in 300 cases, considering the correlation (*r* = 0.3) between aortic size and ASM as described by Bertaux (Bertaux et al., 2007), from which we estimated the period length for retrospective data collection. Quantitative variables are presented as number of cases and prevalence or mean ± standard deviation. The Student's *t*-test and chi-square-test were used to compare group means and categorical variables, respectively. Continuous variables were compared with Pearson's correlation coefficients. The impact of clinically relevant covariates in the main association was investigated in unadjusted and multivariable linear regression models. Intra-reader reproducibility of the study-specific measurements was tested from repeated readings in a randomly selected sample of 20 TEE studies using intraclass correlation coefficients (ICC) and Bland–Altman graphs. All tests were two-tailed and *P-*values < 0.05 were considered statistically significant. The Statistical Package for the Social Sciences (SPSS) software program, IBM, Corp., Armonk, NY, version 23.0 and STATA version 12.0 were used for analysis.

## Results

From the initial 508 studies, we excluded 33 repeated TEE studies performed in the same patients and 14 patients with congenital atrial septal defects, resulting in 461 patients. We also excluded 64 TEE studies without archived images and 61 studies in which images measurements could not be performed in accordance to study protocol, resulting in a final sample of 336 patients. It was noted that 336 patients included in the analysis did not differ from the excluded 125 patients who had TEE during the same study period regarding their age, sex, weight, height, prevalence of atrial fibrillation, or exam indication.

Included patients were 55 ± 16 years old, 49% were women, and 25% were obese (body mass index > 30 kg/m^2^). LVEF <40% was present in 10% of the sample. Previous stroke occurred in 124 (37%) patients and 48 (14.3%) of them had atrial fibrillation. The main indications for TEE were infective endocarditis in 142 (42.2%), stroke in 103 (30.6%) and pre-cardioversion in 44 (13%) patients.

Table 1 shows the echocardiographic measurements and relevant findings. The average diameter of the aortic root was larger in men (35 ± 5 vs. 31 ± 4 mm; *P* < 0.001) and was positively associated with age (*r* = 0.16; *P* = 0.004), height (*r* = 0.35; *P* < 0.001), and weight (*r* = 0.25; *P* < 0.001). Sixty (17.9%) patients had aortic root enlargement (≥38 mm), and three of them had aortic aneurysm (>50 mm). Forty-four percent of the sample had atherosclerotic plaques in the aorta and 50% had some degree of aortic valve fibrocalcific degeneration. The prevalences of ASA and of PFO were 15 and 14%, respectively.

**Table 1:** Transesophageal echocardiographic measurements and findings of patients with clinically indicated studies.

	**Studied sample (*n =* 336)**
**Transesophageal images**	
Aortic root diameter (mm)	33.4 ± 5.1
**Aortic plaques**	
Absent	189 (56)
Simple	99 (30)
Complex	48 (14)
**Aortic valve morphology**	
Normal	178 (49)
Aortic valve sclerosis	145 (40)
Aortic stenosis	38 (10)
Bicuspid aortic valve	5 (1)
Atrial septal diameter (mm)	24.1 ± 7.2
Atrial septal mobility (mm)	3.4 ± 3.7
Atrial septal aneurysm	49 (15)
Patent foramen ovale	46 (14)
**Transthoracic images**	
Left atrial diameter (mm)	42.2 ± 8.0
**Left ventricular ejection fraction**	
<40%	32 (10)
40–50%	26 (7)
>50%	278 (83)
**Diastolic function**	
Normal	118 (35)
Mild dysfunction	113 (34)
Moderate or severe	25 (7)
Indeterminate/non-measurable	80 (24)
Dilated inferior vena cava	64 (19)

*N (%) or mean ± SD*.

**Figure 2:**
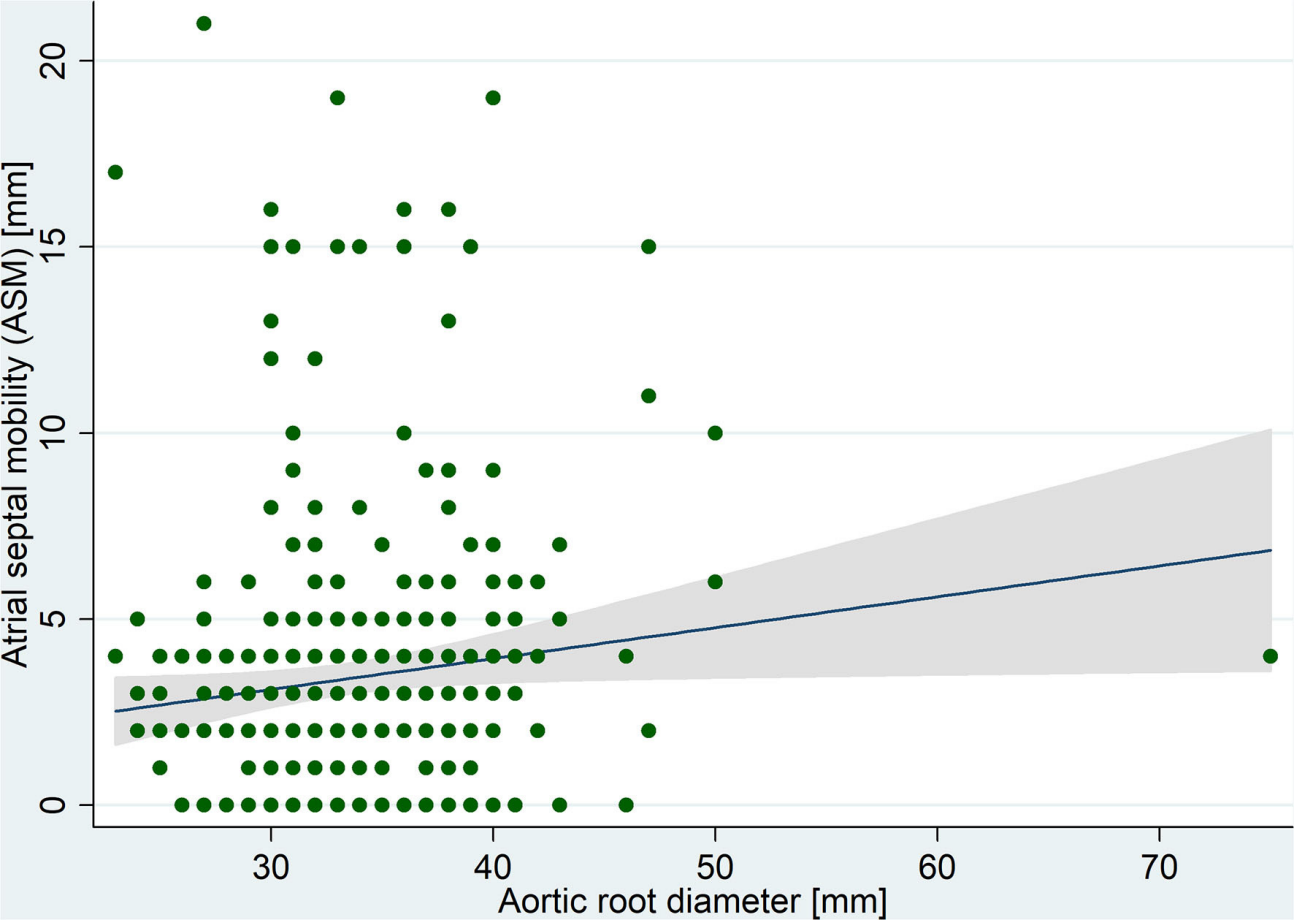
Correlation between atrial septal mobility and aortic root diameter measured in transesophageal echocardiography images.

The mean atrial septal anteroposterior length was 24 ± 7 mm and ASM was measured as 3.4 ± 3.7 mm, ranging from 0 to 21 mm. A significant positive correlation was observed between ASM and aortic root diameter (Figure 2). In multivariable analysis (Table 2), the linear association between ASM and aortic root diameter remained significant when adjusted for age, sex, LV ejection fraction, and left atrial diameter (adjusted *B* coefficient = 0.1; *P* = 0.04). In summary, there was a mean 1 mm increase in ASM for each 10 mm increase in aortic root diameter. No significant linear association was found between aortic size and septal length (*P* = 0.7).

**Table 2:** Crude and adjusted linear regression coefficients between aortic root diameter and atrial septal mobility (ASM).

	***B*** **coefficient**	**CI (95%)**	***P***
**Aortic root diameter**			
Crude	0.08	0.01–0.16	0.03
**Adjusted for:**			
Age	0.08	0.002–0.16	0.04
Sex	0.11	0.03–0.19	0.01
Weight	0.07	−0.01–0.16	0.10
Height	0.08	−0.01–0.17	0.08
LVEF	0.09	0.01–0.17	0.02
Left atrial diameter	0.10	0.02–0.18	0.01
Multivariable adjusted^*^	0.10	0.002–0.2	0.04

* *Adjusted for age, sex, weight, height, left ventricular ejection fraction (LVEF), and left atrial diameter*.

In addition, we were not able to identify significant associations between ASM and the presence of aortic plaques (*P* = 0.61), diastolic dysfunction (*P* = 0.17), or elevated central venous pressure (*P* = 0.37). As expected, patients with ASA had a higher prevalence of PFO (43 vs. 10%; *P* < 0.001). The prevalence of PFO was thrice as frequent among patients with leftward bulging of the atrial septum compared with those with rightward shift (24 vs. 8%; *P* < 0.001).

The reproducibility analysis showed an ICC of 0.91 for aortic diameter, with 95% limits of agreement of −0.9–1.5 mm, and an ICC of 0.87 for ASM, with 95% limits of agreement of −0.2–0.6 mm.

## Discussion

We were able to demonstrate a positive linear association between the aortic root diameter and interatrial septal mobility. Determinants of IAS mobility are less understood, but our findings may suggest new connections between two different well-known ischemic stroke mechanisms. Bertaux et al. demonstrated this same association but in a smaller and stricter sample (Bertaux et al., 2007). Our findings reinforce the hypothesis that, due to their geometry and anatomic proximity, the aortic root dilation could increase ASM. This mechanism was already postulated in case reports of patients with platypnea–orthodeoxia syndrome (Kazawa et al., 2017).

Aortic atherosclerosis and dilation are also associated with cardiovascular risk factors and vascular clinical events (Gardin et al., 2006). Similar to population-based studies, we found a positive linear correlation of aortic diameter with sex, age, weight, and height. Noteworthy, the association between aortic dimension and IAS mobility persisted significant after adjusting for the main determinants of aortic size, reinforcing the independent association between them.

Although patients with aortic plaques had, in average, a larger aorta, we could not find a significant direct association between aortic plaques and augmented ASM. It is worth to say that we found the triple prevalence of PFO in patients with leftward IAS mobility, suggesting that the movement of IAS toward the left atrium could favor the opening of the foramen ovale, probably by increasing the distance between septum primum and secundum.

Methodological limitations of this study design should be considered while evaluating our findings. First, causality could not be inferred due to the cross-sectional design. We acknowledge that our indirect echocardiographic tools are inaccurate compared with invasive measurements to estimate the pressure gradients between atria. The echocardiographic images were analyzed retrospectively and, although the aorta can be easily measured, the widest ASM movement might have been occasionally missed due to the limited set of cine recording. Missing relevant clinical information from retrospectively collected data could mislead associations. It must also be noted that our sample was based on patients of a tertiary hospital, where the prevalence of illness is greater than in population-based studies.

## Conclusion

Our results support the idea that an increased motion of atrial septum can occur due to aortic dilation. There may be a geometric relationship, in which the aortic root manifestations of systemic atherosclerotic disease may partially affect the ASM. These findings raise attention to consider the relevance of aortic root anatomy in the associations between atrial septal characteristics and embolic stroke risk in future clinical studies.

## Data Availability Statement

The raw data supporting the conclusions of this article will be made available by the authors, without undue reservation.

## Ethics Statement

The studies involving human participants were reviewed and approved by GPPG HCPA. Written informed consent for participation was not required for this study in accordance with the national legislation and the institutional requirements.

## Author Contributions

AH contributed to the concept/design, data collection, data analysis/interpretation, drafting of the article. LD'O contributed to the data collection and data analysis/interpretation. EB contributed to the data analysis/interpretation and drafting of the article. MF contributed to the concept/design, data analysis/interpretation and drafting of the article. All authors listed have made a substantial, direct, intellectual contribution to the work, and approved it for publication.

## Conflict of Interest

The authors declare that the research was conducted in the absence of any commercial or financial relationships that could be construed as a potential conflict of interest.

## Publisher's Note

All claims expressed in this article are solely those of the authors and do not necessarily represent those of their affiliated organizations, or those of the publisher, the editors and the reviewers. Any product that may be evaluated in this article, or claim that may be made by its manufacturer, is not guaranteed or endorsed by the publisher.

## Supplementary Material

Supplementary Video 1 A 3D transesophageal echocardiography focused in the aortic valve, illustrating the relationship between the dilated aortic root and the oscillation of the interatrial septum (superior part of the video) during the cardiac cycle.

## References

[B1] Amarenco P. Bogousslavsky J. Caplan L. R. Donnan G. A. Wolf M. E. Hennerici M. G. (2013). The ASCOD phenotyping of ischemic stroke (updated ASCO phenotyping). Cerebrovasc. Dis. 36, 1–5. 10.1159/000352050 23899749

[B2] Bertaux G. Eicher J.-C. Petit A. Dobšák P. Wolf J.-E. (2007). Anatomic interaction between the aortic root and the atrial septum: a prospective echocardiographic study. J. Am. Soc. Echocardiogr. 20, 409–414. 10.1016/j.echo.2006.09.008 17400121

[B3] Chatzikonstantinou A. Krissak R. Schaefer A. Schoenberg S. O. Fink C. Hennerici M. G. (2012). Coexisting large and small vessel disease in patients with ischemic stroke of undetermined cause. Eur. Neurol. 68, 162–165. 10.1159/000339945 22906845

[B4] Chopard R. Meneveau N. (2013). Right-to-left atrial shunting associated with aortic root aneurysm: a case report of a rare cause of platypnea–orthodeoxia syndrome. Heart Lung Circ. 22, 71–75. 10.1016/j.hlc.2012.08.007 22999442

[B5] Di Tullio M. R. (2010). Patent foramen ovale: echocardiographic detection and clinical relevance in stroke. J. Am. Soc. Echocardiogr. 23, 144–155; quiz 220. 10.1016/j.echo.2009.12.008 20152695

[B6] Eicher J.-C. (2005). Hypoxaemia associated with an enlarged aortic root: a new syndrome? Heart 91, 1030–1035. 10.1136/hrt.2003.027839 15761046 PMC1769048

[B7] Gardin J. M. Arnold A. M. Polak J. Jackson S. Smith V. Gottdiener J. (2006). Usefulness of aortic root dimension in persons ≥65 years of age in predicting heart failure, stroke, cardiovascular mortality, all-cause mortality and acute myocardial infarction (from the cardiovascular health study). Am. J. Cardiol. 97, 270–275. 10.1016/j.amjcard.2005.08.039 16442377

[B8] Hasegawa M. Nagai T. Murakami T. Ikari Y. (2020). Platypnoea–orthodeoxia syndrome due to deformation of the patent foramen ovale caused by a dilated ascending aorta: a case report. Eur. Heart J. Case Rep. 4, 1–4. 10.1093/ehjcr/ytaa045 32352045 PMC7180520

[B9] Kazawa S. Enomoto T. Suzuki N. Koshikawa T. Okubo Y. Yoshii S.. (2017). Platypnea-orthodeoxia syndrome in a patient with an atrial septal defect: the diagnosis and choice of treatment. Intern. Med. 56, 169–173. 10.2169/internalmedicine.56.7728 28090047 PMC5337462

[B10] Lamy C. Giannesini C. Zuber M. Arquizan C. Meder J. F. Trystram D.. (2002). Clinical and imaging findings in cryptogenic stroke patients with and without patent foramen ovale: the PFO-ASA Study. Stroke 33, 706–711. 10.1161/hs0302.104543 11872892

[B11] Mas J.-L. Derumeaux G. Guillon B. Massardier E. Hosseini H. Mechtouff L.. (2017). Patent foramen ovale closure or anticoagulation vs. antiplatelets after stroke. N. Engl. J. Med. 377, 1011–1021. 10.1056/NEJMoa1705915 28902593

[B12] Medina A. de Lezo J. S. Caballero E. Ortega J. R. (2001). Platypnea-orthodeoxia due to aortic elongation. Circulation 104, 741–741. 10.1161/hc3101.093603 11489786

[B13] Pearson A. C. Nagelhout D. Castello R. Gomez C. R. Labovitz A. J. (1991). Atrial septal aneurysm and stroke: a transesophageal echocardiographic study. J. Am. Coll. Cardiol. 18, 1223–1229. 10.1016/0735-1097(91)90539-l 1918699

[B14] Reed D. Reed C. Stemmermann G. Hayashi T. (1992). Are aortic aneurysms caused by atherosclerosis? Circulation 85, 205–211. 10.1161/01.cir.85.1.205 1728451

[B15] Saver J. L. Carroll J. D. Thaler D. E. Smalling R. W. MacDonald L. A. Marks D. S.. (2017). Long-term outcomes of patent foramen ovale closure or medical therapy after stroke. N. Engl. J. Med. 377, 1022–1032. 10.1056/NEJMoa1610057 28902590

[B16] Søndergaard L. Kasner S. E. Rhodes J. F. Andersen G. Iversen H. K. Nielsen-Kudsk J. E.. (2017). Patent foramen ovale closure or antiplatelet therapy for cryptogenic stroke. N. Engl. J. Med. 377, 1033–1042. 10.1056/NEJMoa1707404 28902580

[B17] Ward R. P. Don C. W. Furlong K. T. Lang R. M. (2006). Predictors of long-term mortality in patients with ischemic stroke referred for transesophageal echocardiography. Stroke 37, 204–208. 10.1161/01.STR.0000196939.12313.16 16339470

